# Synergistic Effects of Deep Cryogenic and Pulsed Magnetic Field Treatments on the Microstructure and Tensile Properties of Aero-TC4 Titanium Alloy

**DOI:** 10.3390/ma18040817

**Published:** 2025-02-13

**Authors:** Zhijun Ji, Hai Nan, Guirong Li, Shouzuo Guo, Yurong Ye, Hongming Wang, Pengjie Zhou

**Affiliations:** 1 AECC Beijing Institute of Aeronautical Materials, Beijing 100095, China; 2 Advanced Titanium Alloy Precision Forming Technology, Beijing 100094, China; 3 School of Materials Science & Engineering, Jiangsu University, Zhenjiang 212013, China; 4 School of Materials Science & Engineering, Jiangsu University of Science and Technology, Zhenjiang 212003, China

**Keywords:** aero-titanium alloy, deep cryogenic treatment (DCT), pulsed magnetic field (PMF), microstructures and properties

## Abstract

A novel coupled processing method (PDCT) that associated deep cryogenic treatment (DCT) with a high pulsed magnetic field (PMT) was investigated to improve the performance of an as-cast TC4 aero-titanium alloy. Through XRD, SEM, TEM, EBSD, and a properties test, its microstructure and tensile properties and their relationship were investigated. The results show that in comparison with the untreated samples, in the PDCT alloys, the amount of nano-sized precipitates and dislocation density are increased, and this phenomenon is characterized by their combed dislocation morphology. The grain sizes are refined and rounded, and the deformed grains are enhanced, together with the enhancement of low-angle grain boundaries in grains and the transformation from the β phase to the α phase. The (112) crystal orientation is apparently strengthened. The tensile strength, elongation, and fracture energy of the optimized PDCT sample are 921.4 MPa, 7.6% and 5.47 × 10^7^ J/m^3^, which increased by 4.9%, 28.8% and 80.5% compared with the untreated sample, respectively. The tensile fracture exhibits rheological deformation along the phase boundaries. The strength–toughness mechanisms are mainly attributed to the texture, precipitation, dislocation and fine grain strengthening, which stem from the cold contraction and lattice distortion of DCT and the main magneto-plasticity effect of PMT, together with their coupling effects.

## 1. Introduction

The ZTC4 (as-cast Ti-6Al-4V) titanium alloy studied in this paper is an α + β type duplex titanium alloy. It has medium strength and good comprehensive properties and is the most widely used titanium alloy in aircraft [1,2,3,4]. It can be processed into plates, bars and forgings and can also be processed and prepared into castings with complex sizes by precision casting. It is widely used in aircraft and engines, such as casings and mounts in aircraft engine, as well as frames, beams and joints in aircraft, and can reduce the weight of these objects by about 30% when replacing steel.

However, there are several problems in casting using this titanium alloy at present. The first reason is that during the solidification and cooling process of the casting, casting stress will inevitably be generated because of the different cooling rates of each part, the shrinkage resistance, and the volume change caused by the structural transformation [5]. If the casting stress is not relieved, it will remain in the casting as residual stress. Excessive residual stress in castings will cause the deformation of parts in the process of placement, operation, processing, and utilization and a serious reduction in dimensional accuracy, which will then result in cracking [6]. Secondly, the high temperature in the casting process causes coarse grains and other defects, which deteriorate the strength and toughness of the material [7]. Thirdly, as a medium-strength titanium alloy, ZTC4 titanium alloy has many advantages, such as high specific strength, good corrosion resistance, and good stability under low- and high-temperature conditions [8,9,10,11]. However, with the continuously increasing demand for titanium alloys in various high-end manufacturing industries, traditional titanium alloys appear to be a little behind. Therefore, it is necessary to explore a new method of reducing residual stress, promoting grain refinement, and improving the strength and toughness of the ZTC4 alloy to meet gradually growing needs.

Deep cryogenic treatment (DCT) is a kind of solid-state material treatment method in which the sample is placed in a low-temperature field below −150 °C, generally using liquid nitrogen as a low-temperature medium, and the microstructures and properties of the material are improved by adjusting the parameters, such as processing temperature (*T*), treatment time (*t*), cooling rate (*V*), and number of thermal cycles (*N*). At present, besides its specific utilization in chemistry and food storage [12,13], DCT has been applied to improve the properties of ferrous metals and various non-ferrous metals [14]. Li H et al. found that a large number of tiny secondary carbides were precipitated from high-vanadium steel after DCT, and the hardness, impact toughness, and abrasive resistance of the sample were improved [15]. Liu et al. found that the plasticity of HEAp/Al (a high-entropy alloy particle-reinforced Al matrix) composites after DCT for 36 h was greatly improved, and the crack propagation changed from unidirectional to bidirectional [16]. Li et al. argued that low-temperature treatment (DCT) was an effective method of improving the wear resistance and tensile properties of CoCrFeMnNi and reducing its residual stress [17]. Gu et al. studied the effect of DCT on the mechanical properties of a Ti-6Al-4V alloy for biomedical applications. The results show that the Vickers hardness of Ti-6Al-4V increased slightly with the decrease in temperature and increased significantly with the increase in immersion time at −196 °C. It was found that DCT reduced the friction coefficient of Ti-6Al-4V alloy under a 50 N load. In addition, the lower the temperature and the longer the immersion time during DCT, the smaller the friction coefficient will be [18]. Gu et al. also studied DCT alone and DCT followed by aging treatment and found that both could significantly improve the plasticity of a Ti-6Al-4V alloy. After DCT and aging treatment at 650 °C, the ductility of the alloy increased by about 22.7% [19]. M.C. Zang et al. studied the low-temperature tensile properties and deformation behavior of the metastable beta titanium alloy Ti-15Mo-2Al, which has ultra-high tensile strength and intergranular fractures caused by a strong stress concentration at the grain boundary during tension [20]. The studies of the above researchers all found that DCT played a positive role in improving the properties of the alloy.

In recent years, the pulsed magnetic field (PMF) has been introduced into the manufacturing of many new substances [21,22] or to improve material quality [23]. In particular, applying PMF to different alloys can improve the microstructure uniformity of the material, promote dislocation slip, and reduce residual stress [24]. Wang et al. reported that the application of external PMF led to the magneto-plasticity phenomenon of alloyed steel. The rotation of the magnetic domain led to the movement of the magnetic domain wall, disrupting the original balance between the region containing the magnetic domain; the grain was broken, and the change in the internal stress of the grains changed the residual stress in the material [25]. G.R. Li reported that the high-intensity pulsed magnetic field treatment changed the electron spin state between dislocations and obstacles in TiAl-4822 alloy, weakened the pinning effect of obstacles on dislocations, facilitated dislocation movement, and improved the plasticity of materials [26].

In the process of material processing and preparation, it is found that the effect of associated treatment is sometimes more effective than the single treatment [27,28]. Currently, numerous studies have been conducted on the application of single cryogenic treatment and magnetic field treatment in materials science, and the theoretical study of their respective mechanisms has gradually advanced. However, only a few studies have been conducted on an associated treatment method which combines cryogenic treatment with magnetic field treatment. Akhbarizadeh et al. [29] reported that the DCT of tool steels under the action of a static magnetic field changed the distribution of carbides and improved mechanical properties.

In this study, the effects of cryogenic treatment and a high pulsed magnetic field on the microstructure and mechanical properties of the ZTC4 titanium alloy were investigated. This study aimed to optimize the technical parameters of physical field modification treatment and establish the internal relationship between different physical fields, experimental parameters, alloy microstructures, and the mechanical properties of alloys. Additionally, it aimed to explore the optimization mechanism of new technology for the synchronous improvement of strength and toughness.

## 2. Experimental and Procedures

### 2.1. Materials

The original sample of ZTC4 titanium alloy used in the experiment was obtained from the Beijing Institute of Aeronautical Materials, Beijing, China. It was cast in a vacuum consumable electrode arc furnace after melting twice. Table 1 shows the components of the ZTC4 titanium alloy. The casting was treated using a hot isostatic pressing technique by maintaining it at 920 °C for 2.5 h in a 120 MPa argon condition before being cooled to below 300 °C with the furnace in order to eliminate the casting stress and accomplish annealing.

**Figure 1:**
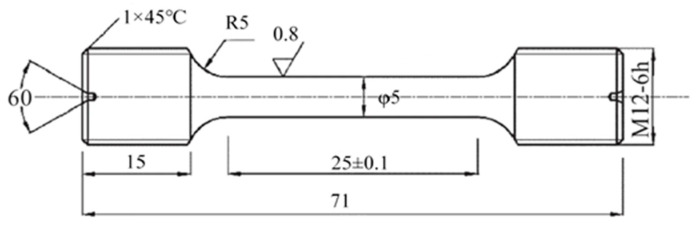
Schematic diagram of the tensile sample (unit: mm).

To test the mechanical properties of the ZTC4 titanium alloy, the material was processed into a standard tensile specimen through precise wire cutting (Figure 1).

### 2.2. Experiment Procedure

To explore the effects of deep cryogenic treatment (DCT) and pulsed magnetic field treatment (PMT) on the mechanical properties and microstructure of ZTC4 titanium alloy, three different modification methods of ZTC4 titanium alloy were designed, as follows.

(1) Deep cryogenic treatment

The liquid DCT method was adopted, which involved direct immersion in liquid nitrogen to maintain the temperature. The used YDS-30-125 liquid nitrogen tank was produced by China Yuxin Aviation Cryogenic Container Co., Ltd., which is located in Xinxiang City, China. The experimental scheme is shown in Table 2. The samples were named according to the cryogenic treatment time (unit: hours) as untreated, DCT12, DCT24, DCT36, and DCT48. The effect of different cryogenic times on microstructure evolution and mechanical properties was investigated to determine the optimized cryogenic treatment time. The cryogenic time was selected based on previous studies [28]. During DCT processing, the ambient temperature was controlled at 25 °C through an air conditioner.

(2) Pulsed magnetic treatment

**Figure 2:**
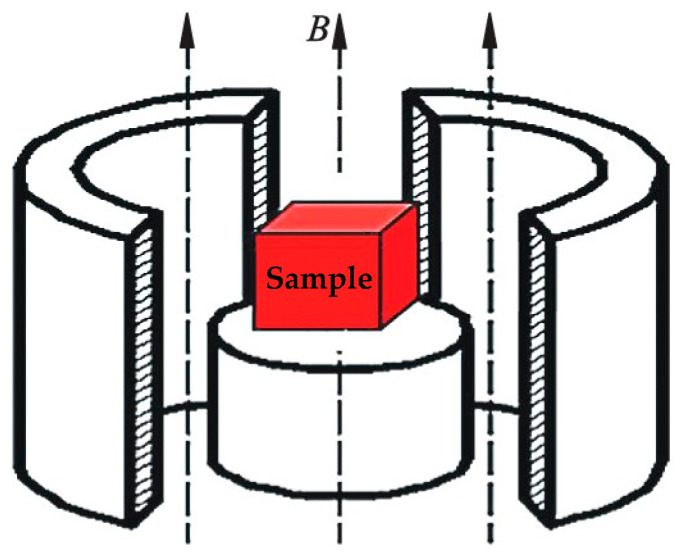
Schematic of the PMT apparatus.

Pulsed magnetic treatment involved placing the sample in the magnetic field coil and uniformly performing the magnetic field treatment on the sample, as shown in Figure 2. The magnetic field generator was produced by the Nanjing Advance Magnetic Components Co., Ltd., which is located in Nanjing City, China, and its type is HSK-H2060. The two most important parameters of the PMF are magnetic induction intensity (*B*) and pulse number (*N*). The differences in *B*, which lead to the deviations in pulse interval, make the characterization of the effective action time of the pulsed magnetic field challenging when using continuous time. Thus, in this experiment, *N* was used to characterize the effective action time of the magnetic field.

The PMT experiment comprised two parts (Table 3); the first was to set *N* = 30 (in accordance with the previous optimization results). Then, *B* was adjusted from 2 T to 5 T, and the corresponding sample numbers were PMT2, PMT3, PMT4, and PMT5. When *B* = B*, the effects of different magnetic field pulse numbers *N* (10, 20, 30 and 40) on the mechanical properties of ZTC4 titanium alloy were investigated, and the optimal pulse numbers N* were obtained by naming the samples PMT10, PMT20, PMT30, and PMT40. Finally, according to the above two parts of the experimental results, the optimal pulse magnetic field parameters were determined. The parameters of pulse magnetic field treatment were selected according to previous studies [26]. During the PMT processing, the input current and voltage were monitored and controlled at an exact value so as to ensure the accuracy of *B*.

(3) PDCT treatment

Building on the results of the previous two studies, the two treatment methods were combined, resulting in PDCT. This involved two steps; at first, the titanium alloy was immersed in the liquid nitrogen for the set soaking times of 12 h, 24 h, 36 h and 48 h. Afterwards, the alloy was removed and exposed to the room-temperature environment. Then, the sample at room temperature was treated with a PMF with *B* = 3 T, *N* = 30. This treatment aimed to investigate the modification effect of associated cryogenic deep treatment and magnetic field treatment on the microstructure and properties of ZTC4 titanium alloy, optimize experimental parameters, and analyze the modification mechanism under the PDCT condition.

### 2.3. Detection Method

An X-ray diffractometer (BRUKER D8 ADVANCE, Bruker, Germany) was used to analyze the phase, and the dislocation density was observed and calculated. The ranges of diffraction angle and scanning speed were set to 20–90°and 5°/min. Field emission scanning electron microscopy (FEI NovaNano 450, Hillsboro, OR, USA), transmission electron microscopy (JEM-2100, Akishima City, Tokyo, Japan), and an electron backscatter diffraction (EBSD) system composed of S3400 field emission scanning electron microscopy (SEM, Tokyo, Japan) and an Oxford prober (London, UK) were used to examine the microstructure evolution of the material before and after treatment, including the changes in grain size, dislocation morphology, phase evolution, and texture. The FEI NovaNano450 SEM had a resolution of 1 nm and an operated acceleration voltage of 10 kV, and the resolution and acceleration voltage of the JEM-2100 transmission electron microscopy (TEM) were 0.1 nm and 250 kV, respectively. The main parameters of EBSD were as follows: a working distance of 30 mm, an acceleration voltage of 3 kV, a beam current of 90 pA, a spatial resolution of 0.05 μm, and an angular resolution of 0.05°. MDI JADE6.5, Digital Micrograph 1.6, and OIM analysis software 8.6 were used to analyze the images or data from X-ray diffraction (XRD), TEM, and EBSD, respectively. The tensile properties of the samples were tested at room temperature using a DDL100 electronic universal testing machine(Changchun Institute of Mechanical Science Co., Ltd., Changchun City, China), and the tensile rate was 1 mm/min.

## 3. Results and Discussion

### 3.1. Performances of DCT and PMT Alloys

Table 4 shows the effect of cryogenic soaking time on the tensile properties of the ZTC4 titanium alloy, and the optimized parameter for liquid cryogenic treatment was ascertained to be 36 h. 

Table 5 shows the tensile properties of the ZTC4 titanium alloy subject to PMT at different *B* and *N* values. The PMT caused various degrees of change in the samples compared with the untreated samples. PMT proved able to achieve simultaneous improvement in strength and toughness, with a more significant effect in the *B* = 3 T, *N* = 30 samples. The ultimate tensile strength and elongation of the sample were 912.5 MPa and 7.3%, showing an increase of 3.9% and 23.7%, respectively, compared with the untreated sample. Therefore, *B* = 3 T and *N* = 30 were the optimal parameters for PMT processing.

### 3.2. Microstructure and Performance of PDCT Alloys

The collaborative processing method was as follows. DCT was processed for 12 h, 24 h, 36 h and 48 h; then, PMF processing with *N* = 30 and *B* = 3 T was performed. The collaborative processing method was abbreviated to PDCT12, PDCT24, PDCT36 and PDCT48 for each sample.

**Figure 3:**
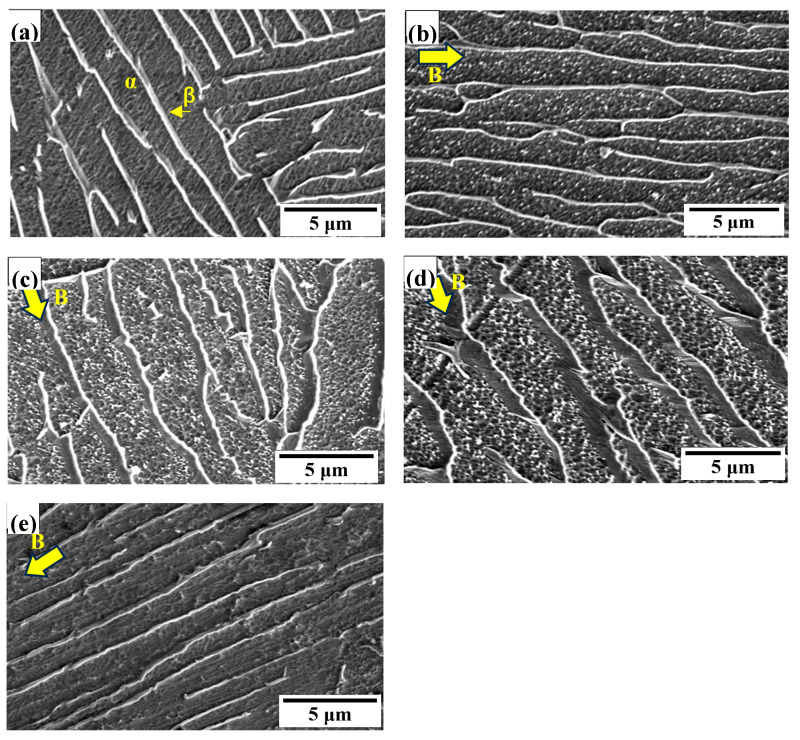
SEM images of PDCT samples with different cryogenic times (8000×). (**a**) Untreated sample; (**b**) PDCT12; (**c**) PDCT24; (**d**) PDCT36; and (**e**) PDCT48.

Figure 3 shows the SEM microstructure (8000×) of PDCT alloys with different DCT times. As shown in Figure 3a, the untreated ZTC4 alloy mainly consisted of a long-stripe α phase with a black color and a needle-like β phase with a white color. No noticeable precipitates were observed in the α phase, and the orientation of the β phase was chaotic.

In the PDCT12 sample (Figure 3b), the grains exhibited orientation characteristics, and small nanoscale precipitates were observed in the α phase. The β phases of PDCT24 and PDCT36 samples exhibited sheet-like morphology, and their height gradually increased along the vertical direction, thus preventing crack propagation under external tensile stress, which was more evident, as shown in Figure 3d. Meanwhile, numerous nanometer precipitates were observed among α phase in the PDCT36 sample. In contrast, the orientation of PDCT48 (Figure 3e) was pronounced, but the small precipitates were not clearly visible. This phenomenon indicates that prolonged DCT is not beneficial for the precipitation process due to stable energy and stress conditions.

Generally, the microstructure of PDCT alloys exhibited the following typical characteristics: first, the number of nano-scale precipitates among the α phase in the PDCT12, PDCT24 (Figure 3c), and PDCT36 samples significantly increased. Second, the morphology of the coarse elongated α phase exhibited the orientation characteristics along the magnetic field direction, as represented by the yellow arrows. Third, the height of the β-phase increased along the vertical direction, which was more pronounced in the optimized PDCT36 sample.

Table 6 shows the comprehensive mechanical properties of the PDCT alloy with different cryogenic times. To ensure the reliability and accuracy of properties, three samples were prepared and processed in the same conditions, resulting in three PDCT12 samples with three corresponding tensile properties. The average property value and standard deviations were calculated based on the three samples.

**Figure 4:**
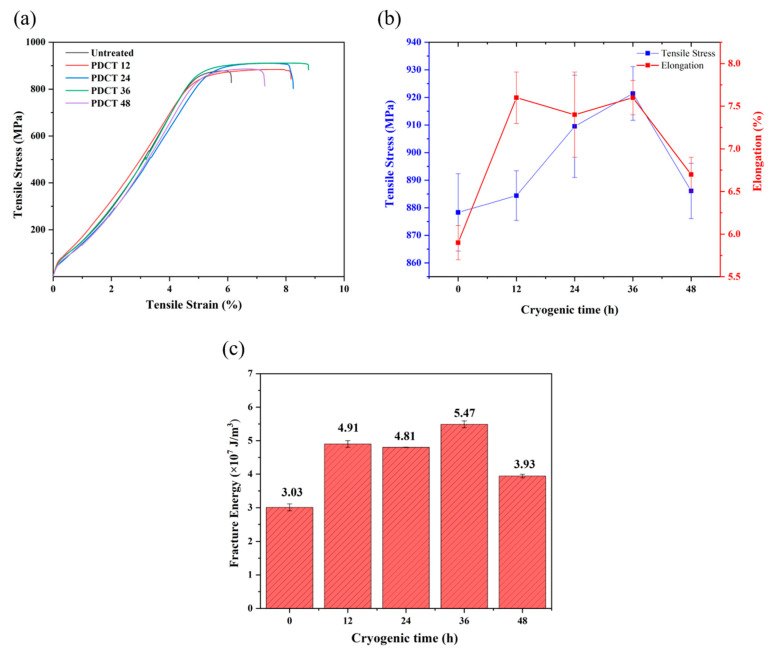
Mechanical properties of PDCT alloy with different cryogenic times. (**a**) Stress–strain curve; (**b**) tensile strength and elongation; and (**c**) fracture energy.

Figure 4a shows the tensile stress–strain curve, and Figure 4b,c show the changes in tensile strength, elongation and fracture energy. Notably, the tensile strength and elongation of PDCT36 were 921.4 MPa and 7.6%, which were 4.9% and 28.8% higher than those of the untreated sample, respectively. The mechanical properties were also superior to those of optimized DCT (soaking for 36 h) or PMF (*B* = 3 T, *N* = 30) samples.

The analysis of the PDCT effect revealed that all the tensile strengths of four PDCT alloys were higher than that of the untreated sample. When the processing time increased from 0 h to 36 h, the tensile strength increased, reached a maximum value at 36 h, and decreased after 48 h. Regarding the change in fracture absorption energy, all the PDCT alloys of fracture absorption energy were superior to that of the untreated sample on the whole. The PDCT36 sample had the highest value of 5.47 × 10^7^ J/m^3^, which was 81% more than that of the untreated sample.

**Figure 5:**
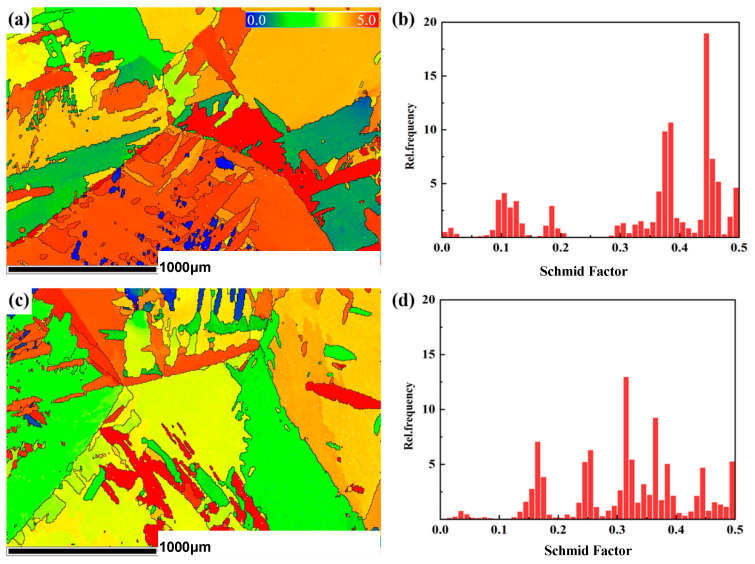
Schmid factor (SF) distribution before and after PDCT: (**a**) SF distribution before PDCT; (**b**) distribution histogram before PDCT; (**c**) SF distribution after PDCT; and (**d**) distribution histogram after PDCT.

The Schmid factor was introduced to investigate the strength characteristic. Figure 5 shows the change and distribution statistics of the Schmid factor before and after PDCT. Figure 5a,c show the distributions of Schmid factors before and after PDCT. The whole Schmid factor of the untreated sample was generally large, with values above 0.3 in most areas. In contrast, the Schmid factor decreased significantly after PDCT processing. According to the statistical results in Figure 5b,d, the Schmid factor of the untreated sample was 0.3465, mainly distributed in the range of 0.35–0.5. The average Schmid factor of PDCT36 was 0.3141 and mainly distributed in the range of 0.3–0.4.

The relationship between the Schmid factor and critical shear stress *τ*_c_ can be expressed as in Equation (1).

(1) $$\tau_{c}=\frac{F}{A}cos\lambda \cdot cos\varphi$$

where *φ* represents the angle between the axial stress (*F*) and the normal of the glide plane whose square is *A*, and *λ* stands for the angle between the force axis and the glide direction. The Schmid factor is defined as cos*λ*·cos*φ.* Because the critical shear stress *τ*_c_ is an inherent property of the material, the magnitude of the Schmid factor cos*λ*·cos*φ* will affect the magnitude of the axial stress (*F*). When the shear stress along the slip direction reaches the critical value, the slip occurs between crystal planes, corresponding to the macroscopic yield limit.

**Figure 6:**
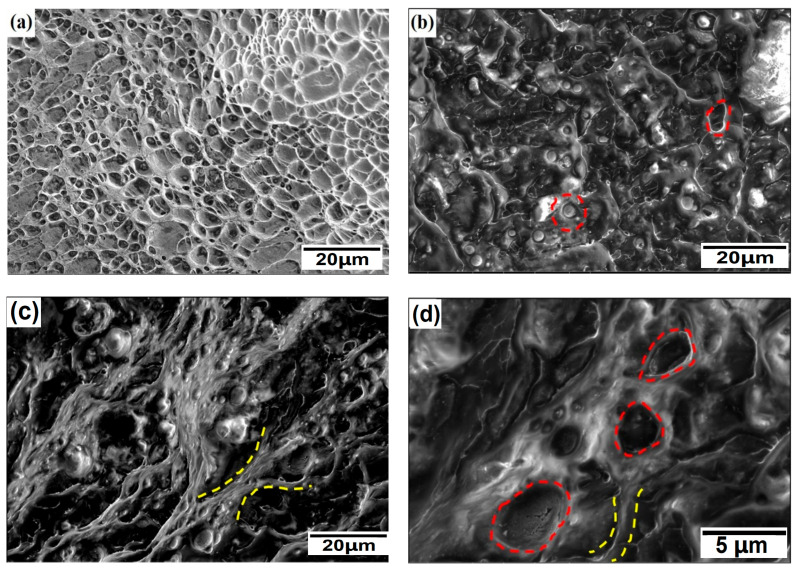
The morphology of the fracture surface for untreated and PDCT samples. (**a**) Untreated sample; (**b**) dimples in PDCT36; (**c**) rheology in PDCT36; and (**d**) magnified dimples and rheology in PDCT36.

Figure 6 shows the tensile fracture of the PDCT sample under SEM. Compared with the untreated sample in Figure 6a, the PDCT sample had noticeable dimples, which are represented by red dashed lines in Figure 6b,d. Figure 6c,d show the rheological characteristics (yellow dashed lines) in the PDCT samples. The fracture surface of PDCT enhanced the toughness and rheological properties of the alloy and supported the performance characteristics in terms of strength and toughness.

In contrast to brittle fracture, ductile fracture was characterized by several features: large energy absorption before actual fracture, large deformation at a certain strain rate, rheological characteristics of metal elongation before fracture, and ductile dimples. According to the characteristics of the elongated microstructure in Figure 6, the alloy deformed along the nano-scaled elongated phase and the phase interface under the action of external forces. The rheological deformation occurred along the phase boundary before reaching the fracture limit. The analysis results showed that as the temperature and cryogenic time decrease and increase, respectively, the lattice distortion will be intensified, and the growth of the precipitated phase will lead to the failure of the fracture originating from the grain interior.

### 3.3. Mechanism Analysis

#### 3.3.1. Structure and Texture

**Figure 7:**
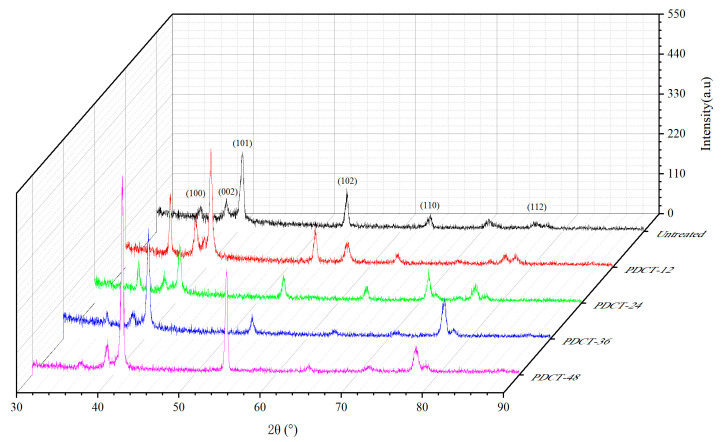
Effect of cryogenic time on the XRD diagram of the PDCT alloy.

Figure 7 shows the X-ray diffraction pattern of the ZTC4 titanium alloy treated by PDCT with different cryogenic times. The peak intensity (*I*) before and after PDCT is shown in Table 7.

The grain orientations of the PDCT alloys significantly changed apparently. The three strongest peaks of the untreated sample corresponded to the (002), (101), and (102) diffraction peaks, among which (101) was the main peak. The three strongest diffraction peaks of PDCT12 and PDCT24 were (100), (101) and (102), respectively. For the PDCT36 and PDCT48 samples, the three strongest diffraction peaks were transferred to the (101), (102) and (112) crystal planes, and the overall intensity significantly decreased.

The texture coefficient was used to analyze the grain orientation change and grain rotation effect. The formula for calculating the texture coefficient of each diffraction intensity peak can be calculated through Equation (2).

(2) $$T_{C}\left(hkl\right)=\frac{I_{i}\left(hkl\right)/I_{0}\left(hkl\right)}{\sum_{i=1}^{n}\left[I_{i}\left(hkl\right)/I_{0}\left(hkl\right)\right]}\times 100\%$$

where *T_C_* (*hkl*) represents the texture coefficient; *I_i_*(*hkl*) is the actual measurement of the peak intensity; *I*_0_(*hkl*) is the peak intensity of the standard crystal face in the PDF card; and *n* represents the number of diffraction peaks. The calculated results are shown in Table 8.

The texture coefficient of PDCT samples changed with different cryogenic times. First, the *T_C_* (*hkl*) of the (100) crystal surface of the ZTC4 sample continued to decrease. The average texture coefficient of four PDCT samples significantly decreased by 48.1% in comparison to the untreated sample. The average texture coefficient of (002) crystal was 11.8% lower than that of the untreated sample. Secondly, the average texture coefficients of the (101) and (102) crystal plane slightly changed. Third, the (112) texture coefficient gradually increased with the processing time and reached a maximum in the PDCT36 sample. Overall, the PDCT weakened the grain orientation in the direction of (100) and (002) but strengthened the grain orientation in the direction of (112), indicating that PDCT treatment promoted grain rotation and orientation, causing some of the grains in the direction of (100) and (002) to be deflected to the direction of (112).

**Figure 8:**
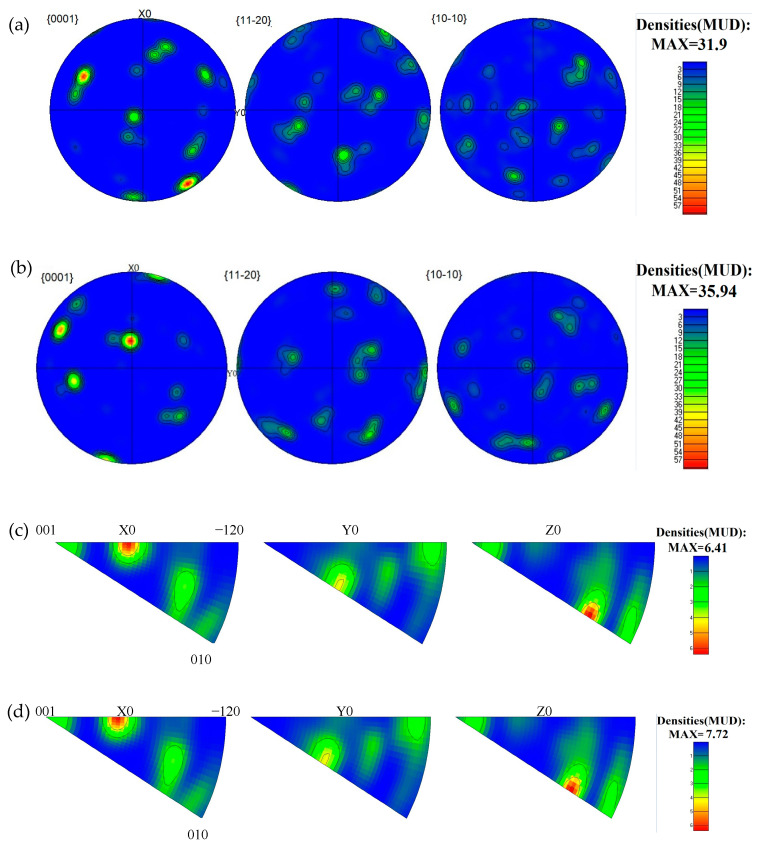
Polar figures of untreated and PDCT samples. (**a**) Polar plot of untreated sample. (**b**) Polar plot of PDCT36 sample. (**c**) Inverse polar plot of untreated sample. (**d**) Inverse polar plot of PDCT36 sample.

To reveal the effect of PDCT on the texture characteristic, the pole and inverse pole figures were tested, as shown in Figure 8. The result revealed the pole figures and inverse pole figures before and after PDCT. High pole density texture caused mechanical anisotropy in the performance of samples because there was a high-density texture in the alloy; thus, the ease of achieving the slip systems required for the initiation of deformation in a certain direction during plastic deformation was different. As a result, differences were observed in the strength and plasticity of the alloy in various directions.

In the pole figures (Figure 8a), the highest strength of the untreated sample was 31.90. After the sample was subjected to PDCT (Figure 8b), the highest strength was 35.94. The plate texture orientation was insignificant. In the inverse pole figure, the PDCT-ed ZTC4 titanium alloy shifted towards (010) in the X0 direction, and the highest strength of the untreated sample’s inverse pole figure was 6.41 (Figure 8c), while after the sample was subjected to PDCT, the highest strength was 7.72 (Figure 8d). The more significant the maximum value of pole density strength, the more pronounced the preferred crystal orientations. The (001) and (−120) directions of some grains in the untreated sample were parallel to the X0 direction of the sample coordinate system, and after PDCT, they shifted towards the (001) and (010) directions. The preferred orientations appeared in these two directions.

#### 3.3.2. Dislocation Strengthening

**Figure 9:**
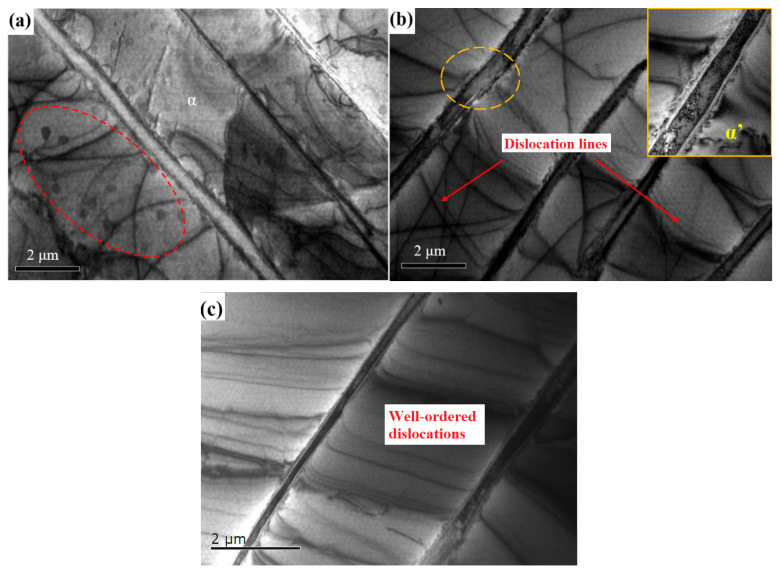
Dislocation characteristic of samples in untreated and PDCT samples. (**a**) Untreated sample; (**b**) proliferative dislocation lines in PDCT36; (**c**) well-ordered dislocations in PDCT36.

Firstly, the morphology of dislocation was focused. Figure 9 shows the dislocation morphology and characteristic phases in the untreated and PDCT samples. Dislocation lines in the untreated sample were few and irregular, which was revealed by the red dotted line (Figure 9a). After PDCT, the dislocation lines in the sample significantly increased, indicating the enhancement of dislocation density (Figure 9b). The high-density dislocation area can act as an obstacle for grains to slip. The dislocation lines in local areas were regular, as shown in Figure 9b,c. These regular lines are a typical structural feature of alloys treated with pulsed magnetic fields, which enhance the mobility of dislocations and the ability to overcome obstacles, thus improving plastic deformation ability.

Then, the full width at half maximum (FWHM, short writing *L*) was introduced to quantificationally characterize the dislocation density. Table 9 shows the *L* values of alloy before and after PDCT treatment. The values in Lines 2–6 are the original data. Combined with the preferred orientation index *T_C_* (*hkl*) (Table 8), the average FWHM value of the alloy (*L*_ave_) can be calculated using Equation (3):

(3) $$L_{ave}=\Sigma L\left(hkl\right)\cdot T_{c}\left(hkl\right)$$

As the cryogenic time increased, the dislocation density initially increased and then decreased. The inflection point was 36 h of cryogenic time. The *L*_ave_ of ZTC4 peaked at PDCT36, indicating the enhancement of dislocation density.

The PDCT processing significantly increased the dislocation density. During the PDCT process, the cast TC4 titanium alloy with a surface area of 1 cm^2^ was subjected to approximately two tons of pressure. The enormous internal stress distorted the crystal lattice and induced dislocations. At the same time, due to the entanglement and pinning of these dislocations, the strength and toughness of the titanium alloy were improved.

Due to the important effects of the geometrically necessary dislocation (GND), the density (*ρ^GND^*) of the alloy was calculated based on EBSD results. A certain conversion relationship was observed between the average local orientation difference (*KAM*_av_) and the geometrically necessary dislocation density (*ρ^GND^*), as expressed in Equation (4) [30].

(4) $$\rho^{GND}=\frac{{2\mathrm{KAM}}_{\mathrm{av}}}{\mu b}$$

where “*μ*” is the scanning step size, “*b*” represents the Burgers vector, and the *KAM* means the local orientation difference. The average *KAM* (*KAM*_av_) can reflect the degree of plastic deformation uniformity, with a higher value indicating greater plastic deformation or higher defect density. Equation (4) can be used to calculate the relative magnitude of geometric dislocation density to determine the stress level during the deformation process.

**Figure 10:**
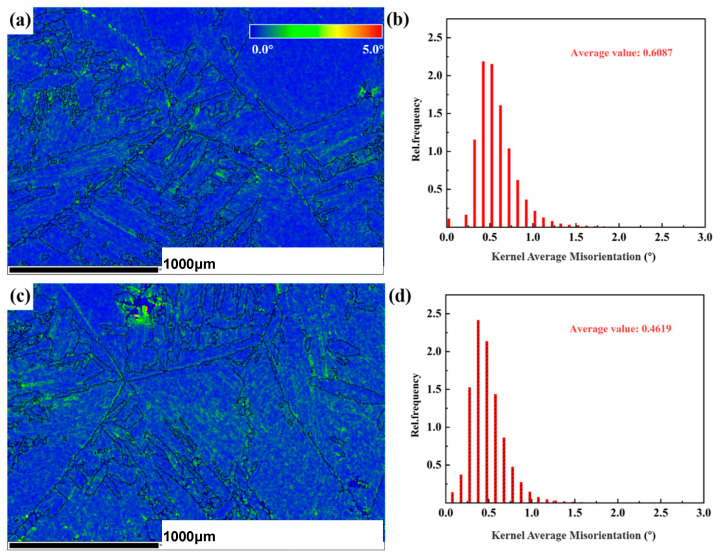
*KAM* diagram and distribution histogram before and after PDCT. (**a**) *KAM* of the untreated sample; (**b**) *KAM* distribution of the untreated sample; (**c**) *KAM* of PDCT36; and (**d**) *KAM* distribution of PDCT36.

Figure 10 reveals the *KAM* diagram and distribution histogram before and after PDCT. Figure 10a,c show the *KAM* distribution maps, where the blue regions represent areas with low local orientation differences and the green regions represent areas with high local orientation differences. Zhang reported that the accumulation of geometrically necessary dislocations at grain boundaries was due to the uneven micro-strain [31].

The greater the difference in local orientation, the higher the cumulative degree of local deformation within the material, resulting in a larger amount of stored deformation energy and a higher geometric density of dislocations (*ρ^GND^*). After the samples were subjected to PDCT, regions with high local orientation differences exhibited a greater overlap with small-angle grain boundaries, providing further evidence for an increase in dislocation density within these localized areas (i.e., dislocation pile-up zones).

Figure 10b,d present the statistical results of the average local orientation difference for the untreated and PDCT samples. Most *KAM*_av_ values for both samples fall within the range of 0°–1°. The *KAM*_av_ of the untreated sample was 0.6087 and decreased to 0.4619 in PDCT36. Although the *KAM*_av_ of the PDCT36 sample decreased, some local areas had high *KAM* values, represented by a bright green color, as shown in Figure 10c. This phenomenon, marked by the concentration of local orientation difference, corresponds to an increase in the geometric density of dislocations within certain regions.

**Figure 11:**
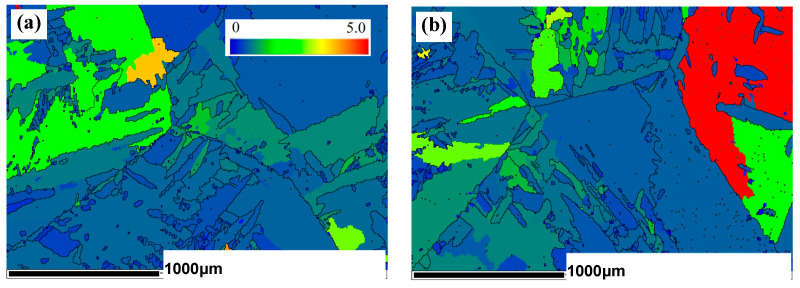
GOS before and after PDCT. (**a**) Untreated; (**b**) PDCT36.

The grain orientation spread (GOS) of the cross-section of untreated and PDCT samples was studied, as shown in Figure 11. The GOS diagram is regarded as a measurement of dislocation density and strain in a single grain. Its average value is a quantitative estimate of the orientation deviation within the grain, mainly reflecting some grains with large deformation. The color bar is displayed in the upper right location of Figure 11a. The region with a small GOS indicates a more uniform strain distribution, while a large GOS represents a higher dislocation density, which may be caused by dislocation plugging. The red part in the upper right corner of Figure 11b shows the region with the highest GOS value compared with other grains, which is close to 5.0. This value shows that the average orientation difference in this grain is large, indicating a higher dislocation density and higher strain.

**Figure 12:**
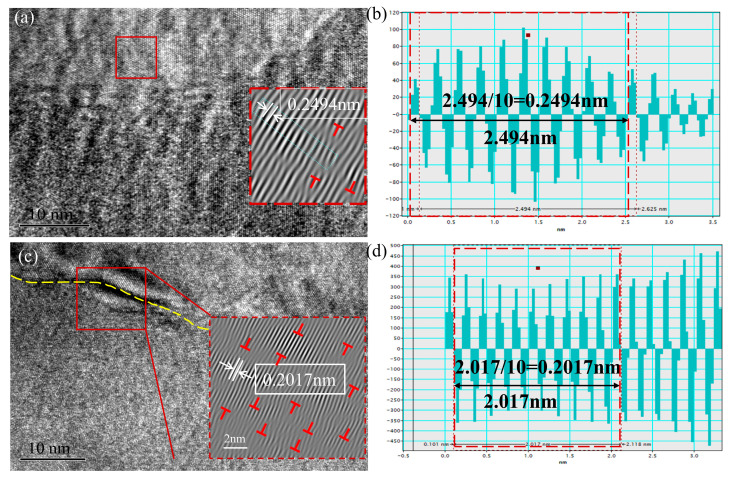
IFFT images of high-resolution and lattice spacing of untreated and PDCT samples; (**a**) high-resolution image of untreated sample; (**b**) lattice spacing of untreated sample; (**c**) high-resolution image of PDCT36 sample; (**d**) lattice spacing of PDCT36 sample.

The high-resolution TEM used to examine the dislocation characteristic in typical samples. Figure 12 shows the high-resolution IFFT (inverse fast Fourier transform) images of the untreated and PDCT samples. The T symbols refer to the dislocation location. The yellow dotted line in Figure 12c meant an apparent deforming line. The corresponding average lattice spacings were 0.2494 nm and 0.2017 nm, respectively. The decrease in the lattice spacing implies a stronger interaction between atoms or ions in the crystal, which improves physical properties.

#### 3.3.3. Precipitation Strengthening

**Figure 13:**
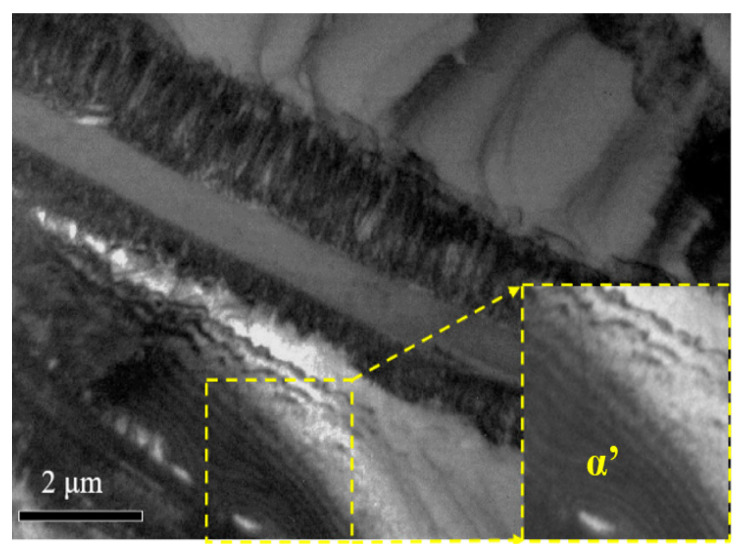
Precipitations in the PDCT alloy.

As shown in Figure 13, some secondary phases appeared along the phase interface between α and β in the PDCT sample. The α’secondary phase appeared at the β edge, demonstrating the transition from β to α. Therefore, the α lath was thickened after PDCT, which was also considered to be the result of the incomplete phase transition promoted by PDCT. The secondary α′ phase was short and rod-like at the micro and nanoscale (as shown by the yellow lines in Figure 9b). As shown in Figure 9a, there were a few second precipitates in the untreated sample.

**Figure 14:**
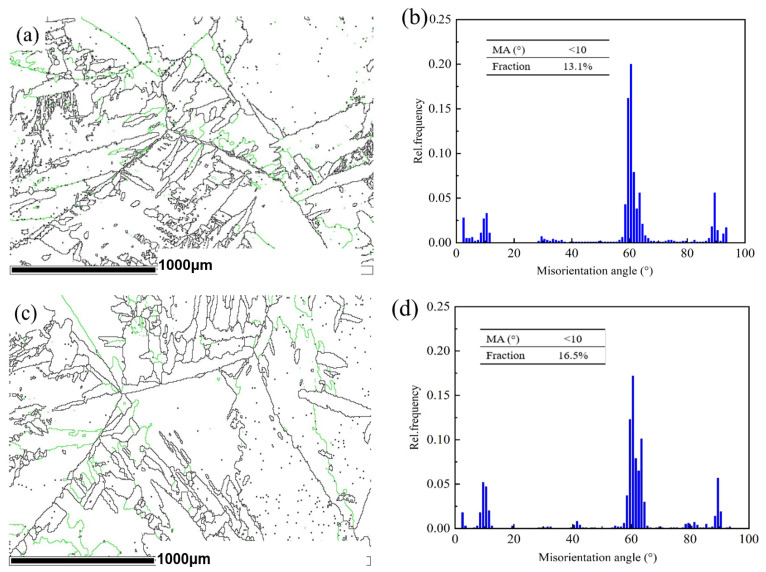
Type and orientations of grain boundaries (GB) in untreated and PDCT36 samples: (**a**) GB type of untreated sample; (**b**) GB orientations of untreated sample; (**c**) GB type of PDCT36 sample; (**d**) GB orientations of PDCT36 sample.

Because the secondary α′ phase mainly appeared at the grain boundary, the types and orientations of the grain boundaries of the untreated and PDCT samples were investigated, as shown in Figure 14. The black lines represent high-angle grain boundaries (HAGBs), while the green lines represent low-angle grain boundaries (LAGBs) whose misorientation angle (MA) is less than 10°. The black HAGBs were the main component in the two typical samples. In the untreated sample, the green LAGBs were initially preferentially distributed at the grain boundaries (Figure 14a).

As shown in Figure 14b,d, the percentage of LAGBs in the untreated sample was 13.1%, while the percentage of those in PDCT36 increased to 16.5%. SEM microstructure analysis revealed that the phase transformation occurred from the β phase to the α′ phase in some locations where the grain boundaries were less than 10° during the PDCT. The driving energy of phase transformation in the LAGB area was the energy stored in the internally accumulated dislocations. In addition, because of the Burgers orientation relationship between the β and α phases, a single β phase can be transformed into twelve different α variants [32]. This transformation means that various selections will occur during the PDCT process, resulting in the precipitation of many secondary α′ phases with small-angle grain boundaries on both sides of the α and β grain boundaries.

#### 3.3.4. Fine Grain Strengthening

**Figure 15:**
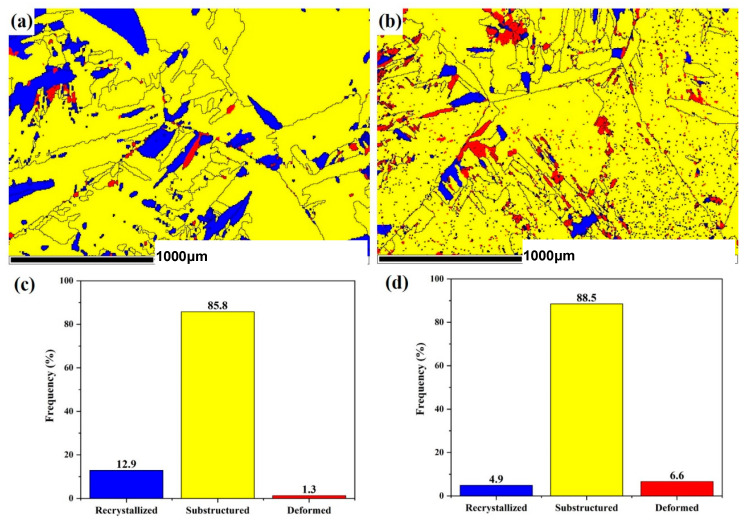
Comparison of grain morphology and type changes before and after PDCT. (**a**) Grain morphology of untreated sample; (**b**) grain morphology of PDCT36 sample; (**c**) grain type of untreated sample; (**d**) grain type of PDCT36 sample.

We initially focus on the grain type in typical TC4 titanium alloys (Figure 15). As shown in Figure 15a,b, the blue, yellow, and red regions represent recrystallized grains, sub-grains, and deformed grains, respectively. Figure 15c,d show the proportion of different grains. After PDCT treatment for 36 h, the recrystallized grains of the samples were reduced from 12.5% to 4.9% and transformed into sub-grains (increasing from 85.8% to 88.5%) or deformed crystals (increasing from 1.3% to 6.6%). The percentage of sub-grains and deformed grains increased by 2.7% and 5.3% in PDCT36 compared with the untreated sample. This phenomenon indicates that PDCT treatment promotes the nucleation and growth of sub-grain structures, mainly through aggregation at grain boundaries or phase boundaries, transforming into small-sized sub-grains driven by high-density dislocation.

The enhancement of deformed grains indicates that the microplastic deformation of ZTC4 alloy samples is induced under the cryogenic condition and PMF. Liu et al. used the Volterra dislocation model of the continuum to calculate the strain energy of edge dislocation under the influence of the external magnetic field by applying elastic theory and electrodynamics theories. The results showed that the strain energy of paramagnetic edge dislocation increased with an increase in magnetic field [33]. The increase in dislocation energy affected the dislocation unpinning, resulting in plastic deformation. The growth of deformed grains indicated that the PDCT sample was subjected to micro-plastic deformation, and some deformed grains reached detectable size, indicating that the PDCT alloy exhibited a significant “magneto-plastic” effect. Due to the other causes of plastic deformation, the magneto-plastic effect varied with crystal orientation. The magnetic field reduced the micro-strain around dislocation lines, which was induced by the cold contraction stress field, making dislocations easier to move. This process increased the slip line spacing and the complete deformation of the alloy [34,35].

**Figure 16:**
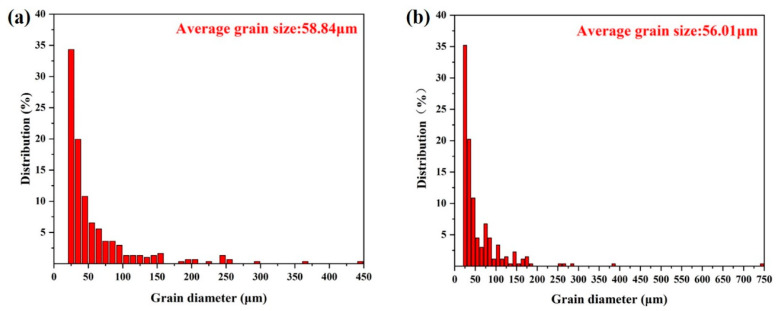
Grain size distribution before and after PDCT: (**a**) untreated sample; (**b**) PDCT36 sample.

We now focus on the distribution of grain size before and after PDCT (Figure 16). After the sample was subjected to PDCT, the average grain size decreased from 58.84 μm to 56.01 μm. The general trend of grain size reduction shifted from the columnar distribution to the left, and the number of grains larger than 100 μm in diameter significantly decreased. When the grain size was smaller, the grain boundary area became larger. Nucleation at grain boundaries was more likely to occur during solid-phase transformation because of the high energy of the grain boundaries and the significant activity of atoms. The increase in the number of grain boundaries with the refinement of grains enhanced the nucleation rate, facilitating the generation of the precipitated phase to a certain extent. The overall grain size refinement means the simultaneous improvement of strength and toughness at the macro level.

#### 3.3.5. Discussion

Overall, the combination of cryogenic treatment and magnetic field treatment effectively improved the tensile properties. The main factors contributing to the strengthening and toughening mechanism are summarized as follows: (1) optimized morphology of α and β phase; (2) preferred grain orientation along (112); (3) high-density dislocation and compact lattice spacing; (4) nano-sized and uniformly distributed precipitates; and (5) dominant sub-grains and deformed grains together with grain refinement. Table 10 summarizes the main microstructural characteristics and performance of the untreated and optimized PDCT36. The corresponding strengthening and toughening mechanism can be found in the right column.

The combined effects of cryogenic treatment and PMF are analyzed as follows.

First, the cryogenic treatment resulted in cold shrinkage stress and lattice distortion. The changes in the grain accelerated the moving ability of dislocation, leading to dislocation plugging and increased dislocation density. Under the continuous effect of cold shrinkage force, the dislocation moved in the polycrystal, and the grain near the grain boundary increased the slip resistance due to differences in local orientation. As a result, dislocation movement was hindered, making it difficult for dislocations to cross grain boundaries, leading to their accumulation at the boundaries. The high-density dislocation formed entanglements, increasing strength through the dislocation-strengthening mechanism.

After cryogenic treatment, the dislocations that accumulated at grain boundaries formed a dislocation wall. In the subsequent magnetic field treatment, the magnetic field facilitated the release of stress that improved the transformation of recrystallized grains existing in the as-cast alloy. At this point, the dislocation at the grain boundary began to diffuse at a high speed. Some unstable dislocations were released during the diffusion process, rearranging to form a large angle orientation, that is, forming a small sub-structured grain. Figure 15b shows some fine grains. As shown in Figure 15d, the percentage of sub-structured grains increased from 85.8% in the untreated sample to 88.5% in the PDCT sample. The whole grain size reduced from 58.84 μm to 56.00 μm. According to the Hall–Petch equation, the refined grains imparted higher strength to the material [36]. The grain refinement significantly reduced the impurity content of unit grain boundary area segregation. Meanwhile, during the process of magnetic field treatment, the magnetic field reduced stress concentration, which facilitated the precipitation of the nanoscale secondary alpha phase [37].

## 4. Conclusions

In this study, a novel coupled processing method combining deep cryogenic treatment (DCT) and high PMT was used to enhance TC4 titanium alloys. The mechanism of microstructure and performance improvement was analyzed. The following findings were obtained.

(1) The tensile strength, elongation and fracture absorption energy of the optimized PDCT sample were 921.4 MPa, 7.6%, and 5.47 × 10^7^ J/m^3^, respectively, showing increases of 4.9%, 28.8% and 80.5% compared with the untreated sample. The tensile fracture exhibited rheological deformation along the phase boundaries.
(2) The grain orientation of the PDCT alloy significantly changed, thus exhibiting different textural characteristics, and some grains in the direction of (002) and (100) were deflected to the direction of (112). The polar density and intensity in the polar and inverse polar diagrams of the treated samples were higher than those of the untreated samples, indicating that the PDCT increased the preferred orientation characteristics of the crystals. In this case, PDCT acted as the texture strengthener.
(3) The cryogenic treatment improved the dislocation density and formed a dislocation wall at the grain boundary due to the cold shrinkage force. The PMF facilitated the dislocation rearrangement and improved the moving ability of the dislocation, making PMF the dislocation strengthener.
(4) The secondary nano α′ phase with a width of >100 nm appeared at the edge of the β phase. The proportion of grain boundary with small angle increased from 13.6% to 16.5%, which facilitated the transformation of β to the α and α′ phases. In the PDCT sample, the long-strip β phase had good grain connectivity and exhibited orientation characteristics along the magnetic field direction, thus increasing vertical β phase height. This phenomenon indicates that PDCT acts to strengthen the precipitated phase.
(5) PDCT facilitated the transformation of recrystallized grains into sub-crystalline and deformed grains. Meanwhile, the average grain size decreased due to the generation of nanoscale sub-crystalline grains. PDCT decreased the lattice spacing and increased the interatomic forces. Thus, it acted to strengthen the refined crystals.

## Figures and Tables

**Table 1:** Components of the ZTC4 titanium alloy (wt.%).

Element	Ti	Al	V	Fe	Si	C	N	H	O
	Base	5.5~6.8	3.5~4.5	0.30	0.15	0.10	0.05	0.015	0.20

**Table 2:** Scheme for DCT experiments using ZTC4 titanium alloy.

Specimens	Cryogenic Temperature *T* (°C)	Cryogenic Time *t* (h)
DCT12	−196	12
DCT24	−196	24
DCT36	−196	36
DCT48	−196	48

**Table 3:** Scheme for PMT experiment.

Samples	Magnetic Induction Intensity *B* (T)	Pulses Counts *N*
Untreated	0	0
PMT2	2	30
PMT3	3	30
PMT4	4	30
PMT5	5	30
PMT20	3	20
PMT30	3	30
PMT40	3	40

**Table 4:** Tensile properties of the DCT-ed ZTC4 titanium alloy using different cryogenic times.

Samples	Cryogenic Temperature (°C)	Cryogenic Time (h)	Tensile Strength, *σ*_b_ (MPa)	Elongation, *δ* (%)	Remarks
Untreated	Room temperature	0	878.3	5.9	
DCT12	−196 °C	12	859.2	7.3	
DCT24	−196 °C	24	893.2	7.5	
DCT36	−196 °C	36	916.5	7.5	Best
DCT48	−196 °C	48	875.9	6.8	

**Table 5:** Mechanical properties of the PMT-ed ZTC4 titanium alloy.

Samples	Magnetic Induction Intensity, *B* (T)	Pulse Count *N*	Tensile Strength, *σ*_b_ (MPa)	Elongation, *δ* (%)	Remarks
Untreated	/	/	878.3	5.9	Best *B*
PMT2	2	30	901.6	6.6	
PMT3	3	30	912.5	7.3	
PMT4	4	30	910.4	7.1	
PMT5	5	30	897.1	6.9	
PMT20	3	20	901.9	7.2	
PMT30	3	30	912.5	7.3	Best *N*
PMT40	3	40	899.3	6.9	

**Table 6:** Mechanical properties of PDCT samples with different cryogenic times.

Samples	Tensile Strength (MPa)	Standard Deviations (SD) of Tensile Strength (MPa)	Elongation (%)	SD of Elongation	Fracture Energy (J/m^3^)	SD of Fracture Energy (J/m^3^)
Untreated	878.3	2.1	5.9	0.3	3.03 × 10^7^	0.09 × 10^7^
PDCT12	884.4	1.7	7.6	0.1	4.91 × 10^7^	0.11 × 10^7^
PDCT24	910.5	1.6	7.4	0.1	4.81 × 10^7^	0.08 × 10^7^
PDCT36	921.4	1.7	7.6	0.2	5.47 × 10^7^	0.12 × 10^7^
PDCT48	886.1	2.0	6.7	0.1	3.93 × 10^7^	0.11 × 10^7^
Average of PDCT samples (AVE)	900.6	/	7.3	/	4.78 × 10^7^	/
AVE vs. Untreated	Increase of 2.5%	/	Increase of 23.7%	/	/	Increase of 57.8%

**Table 7:** The relative peak intensities (*I*) of the main strong peaks before and after PDCT.

*I* (%)	(100)	(002)	(101)	(102)	(112)
Untreated	17.2	25.9	100.0 *	48.9	8.0
PDCT12	58.6	30.2	100.0	32.1	7.1
PDCT24	62.6	27.0	100.0	49.6	32.2
PDCT36	10.7	11.9	100.0	17.7	37.4
PDCT48	2.1	6.6	100.0	33.3	7.1

Note: * The intensity of the strongest (101) peak is set to 100%. The peak intensities of other peaks are calculated on the basis of this and are therefore named relative peaks.

**Table 8:** The *T_C_* (*hkl*) of main crystal planes for PDCT alloys with different cryogenic times (unit: %).

Samples	*T_C_* (100)	*T_C_* (002)	*T_C_* (101)	*T_C_* (102)	*T_C_* (112)
PDCT12	45.87	16.38	14.05	9.22	12.47
PDCT24	33.95	9.72	9.33	9.46	37.54
PDCT36	8.74	6.45	14.05	5.08	65.67
PDCT48	4.15	8.65	33.95	23.12	30.13
Untreated	47.64	13.68	18.03	10.98	12.31
Average of PDCT samples (AVE)	23.67	10.31	17.85	11.72	36.45
AVE. vs. Untreated	Decrease of 48.1%	Decrease of 11.8%	Almost unchanged	Almost unchanged	Increase of 196.1%

**Table 9:** The FWHM of alloy before and after PDCT treatment.

Samples	*L*(100)	*L*(002)	*L*(101)	*L*(102)	*L*(112)	$L_{ave}$
Untreated	0.34	0.39	0.41	0.39	0.45	1.98
PDCT12	0.27	0.38	0.35	0.43	0.56	1.98
PDCT24	0.27	0.39	0.39	0.46	0.54	2.04
PDCT36	0.39	0.42	0.38	0.42	0.49	2.11
PDCT48	0.43	0.38	0.30	0.33	0.53	1.97

**Table 10:** Summary and comparison of main microstructural characteristics and performance.

Items	Untreated	PDCT36	Mechanism
Precipitates within the α phase	none	Massive nano-sized precipitates	Precipitation strengthening
α morphology	Random	Complete and orientated	α phase strengthening
β morphology	Striped	Vertical preference growing	β phase strengthening
Grain type	More recrystallized grains	Sub-grains and deformed grains increase	Fine-grain strengthening
Grain size	58.84 μm	56.00 μm	Fine-grain strengthening
Grain boundary	HGAB occupies	LGAB occupies	Dislocation strengthening Precipitation strengthening
Dislocation density	Low	High	Dislocation strengthening
*KAM*	0.61	0.46	Decrease in average while increase in local areas
Lattice spacing	2.494 nm	2.017 nm	Fine-grain strengthening
Texture	Orientated planes are (100) and (002)	Orientated planes are (112)	Texture strengthening
Schmid factor	0.3465	0.3141	Yield strength increases
Residual stress	−577 MPa	−480.3 MPa	Stability increase

## Data Availability

The data presented in this study are available on request from the authors due to privacy.
